# Adverse Childhood Experiences, Gender Identity and Substance Misuse: Challenges in Addiction Psychiatry – A Case Report

**DOI:** 10.1192/j.eurpsy.2025.979

**Published:** 2025-08-26

**Authors:** M. D. Pereira, P. Carriço

**Affiliations:** 1 Psiquiatria da Infância e da Adolescência, Unidade Local de Saúde de Coimbra; 2 Centro de Respostas Integradas de Coimbra, ICAD, Coimbra, Portugal

## Abstract

**Introduction:**

Adverse Childhood Experiences, referring to potentially traumatic events such as sexual abuse, rejection, and family dysfunction, are strongly associated with the development of psychiatric disorders, including anxiety, depression, and substance misuse. Peer victimization related to sexual orientation and gender identity or expression has been linked to higher rates of depressive symptoms, disrupted educational paths, trauma, and increased use of substances such as alcohol and drugs. Gender-diverse youth are particularly vulnerable to substance misuse, especially in environments with limited support and prevalent discrimination.

**Objectives:**

To emphasize the complex interactions between substance use, early trauma, and gender identity struggles, while highlighting the importance of a multidisciplinary therapeutic approach.

**Methods:**

This case report was developed through detailed psychiatric interviews and assessments conducted during the patient’s follow-up in an outpatient addiction treatment unit. The case description is supported by a focused literature review on PubMed using the keywords “Adverse Childhood Experiences”, “Substance Use Disorder” and “Gender Identity”.

**Results:**

The patient is a young adult with a history of polysubstance use, starting in adolescence. His substance use was closely associated to early trauma, including childhood sexual abuse and rejection by family and peers after disclosing his sexual orientation. These events led to dropping out of school, running away from home, and experiencing homelessness. As a result, he developed long-standing anxiety, depressive symptoms, and emotional instability. Internal struggles with gender identity, alongside ongoing familial conflict and unstable romantic relationships, further worsened his psychopathology. After a multidisciplinary intervention involving psychiatry, psychology, and social services, he showed significant improvements in emotional regulation, anxiety management and has remained abstinent since starting treatment.

**Conclusions:**

Personal experiences, especially during childhood and adolescence, profoundly influence mental health and behaviors in adulthood. This case underscores the interaction between adverse childhood experiences, substance misuse, and discrimination related to sexual orientation, illustrating how these factors collectively impact psychiatric health. The patient’s journey through addiction, identity struggles, and mental health challenges reflects the deep influence of both personal trauma and systemic issues. This case highlights the need for multifaceted therapeutic approaches that address the psychological, social, and familial factors underlying the patient’s condition.

**Disclosure of Interest:**

None Declared

